# Principal Component Analysis of the Running Ground Reaction Forces With Different Speeds

**DOI:** 10.3389/fbioe.2021.629809

**Published:** 2021-03-25

**Authors:** Lin Yu, Qichang Mei, Liangliang Xiang, Wei Liu, Nur Ikhwan Mohamad, Bíró István, Justin Fernandez, Yaodong Gu

**Affiliations:** ^1^Loudi Vocational and Technical College, Loudi, China; ^2^Faculty of Sports Sciences and Coaching, Sultan Idris Education University, Tanjong Malim, Malaysia; ^3^Faculty of Sports Science, Ningbo University, Ningbo, China; ^4^Research Academy of Grand Health, Ningbo University, Ningbo, China; ^5^Auckland Bioengineering Institute, University of Auckland, Auckland, New Zealand; ^6^Faculty of Engineering, University of Szeged, Szeged, Hungary; ^7^Department of Engineering Science, University of Auckland, Auckland, New Zealand

**Keywords:** gait biomechanics, gender difference, machine learning, running velocity, ground reaction force

## Abstract

Ground reaction force (GRF) is a key metric in biomechanical research, including parameters of loading rate (LR), first impact peak, second impact peak, and transient between first and second impact peaks in heel strike runners. The GRFs vary over time during stance. This study was aimed to investigate the variances of GRFs in rearfoot striking runners across incremental speeds. Thirty female and male runners joined the running tests on the instrumented treadmill with speeds of 2.7, 3.0, 3.3, and 3.7 m/s. The discrete parameters of vertical average loading rate in the current study are consistent with the literature findings. The principal component analysis was modeled to investigate the main variances (95%) in the GRFs over stance. The females varied in the magnitude of braking and propulsive forces (PC1, 84.93%), whereas the male runners varied in the timing of propulsion (PC1, 53.38%). The female runners dominantly varied in the transient between the first and second peaks of vertical GRF (PC1, 36.52%) and LR (PC2, 33.76%), whereas the males variated in the LR and second peak of vertical GRF (PC1, 78.69%). Knowledge reported in the current study suggested the difference of the magnitude and patterns of GRF between male and female runners across different speeds. These findings may have implications for the prevention of sex-specific running-related injuries and could be integrated with wearable signals for the in-field prediction and estimation of impact loadings and GRFs.

## Introduction

Ground reaction force (GRF) has been a key and useful parameter in biomechanics (Munro et al., 1987), including GRF in the vertical, anterior–posterior (ant–post), and medial–lateral (med–lat) directions recorded from a three-dimensional force plate. This parameter was used to investigate the human movement and gait patterns (Wannop et al., 2012; Horsak et al., 2020), athletic performance (Colyer et al., 2019), impact loadings (Mei et al., 2019), abnormal musculoskeletal conditions (Hendry et al., 2020; Williams et al., 2020), and orthotics evaluation (Logan et al., 2010; Soares et al., 2016). Thus, understanding the variations of GRF overtime could inform movement analysis and impact stress from the vertical GRF or shear stress from the resultant GRFs.

Typically, the vertical GRF consists of a first peak as heel landing and a second peak during pushing-off (Wannop et al., 2012; Mei et al., 2015), and the ant–post GRF includes braking peak and propulsion peak (Wannop et al., 2012). In contrast, the med–lat GRF showed little consistency and high variations in the pattern and shape, which might be due to the different foot placements, footwear condition, and environmental surface while running (McClay and Cavanagh, 1994; Samaan et al., 2014; Seeley et al., 2020). Accordingly, the studies on the GRFs were mainly conducted to analyze the vertical and med–lat aspects of GRF, investigating the biomechanical loadings with different running footwear (Logan et al., 2010), risk of running-related injuries (van der Worp et al., 2016), athletic performance during a sprint (Colyer et al., 2019), and biomechanical response to fatigue between sexes (Bazuelo-Ruiz et al., 2018).

As documented in the literature, factors such as speed, sex, fatigue, and footwear altered the response in the running GRFs (Logan et al., 2010; Mei et al., 2015; Bazuelo-Ruiz et al., 2018). Female runners presented a reduced loading rate (LR) and first peak, whereas males had higher peak propulsive forces under fatigue conditions (Bazuelo-Ruiz et al., 2018). The different footwear may also redistribute the impact peak forces and propulsion forces (Logan et al., 2010). While running with different speeds, the peak GRFs were not always linearly correlated with body weight (BW) *via* normalization with BW times height and BW times leg length (Wannop et al., 2012), particularly observed in the first peak of vertical ground reaction force (vGRF) across different speeds (Seeley et al., 2020). The loadings of faster or slower running differed from preferred running velocity on a treadmill (Kluitenberg et al., 2012). However, the analyses mentioned earlier mainly focused on discrete values at a certain time point in the dataset. Similar to other biomechanical parameters (joint angles, joint moments, and muscle activities), the GRF varies over time during stance. Thus, approaches on advanced or time-varying statistics could be used to decipher variations between sexes and across different speeds.

Recently, several advanced statistical techniques, such as the principal component analysis (PCA) and functional data analysis (FDA), were developed and used to conduct statistical modeling for time-varying data in biomechanics. The PCA modeling is a multivariate technique to reduce the high-dimensional data matrices into orthogonal principal components (PCs), which explains major modes of variances within the dataset (Deluzio et al., 1997; Lever et al., 2017; Cushion et al., 2019). Each mode of variation reported in the PCA modeling is a key feature extraction, which would be applied in the machine learning technique (Phinyomark et al., 2018). Whist, the FDA modeling treats the whole time-series dataset as a function defined as a finite discrete timepoint (Ullah and Finch, 2013). A similar statistical technique with PCA in the FDA is the functional PCA, which treats the biomechanical data as a function, not a series of individual numbers and smoothed before PCA modeling. Both PCA and functional PCA presented similar results while accounting for the variations in the dataset of gait kinematics (Warmenhoven et al., 2021). A further explicit variance of each PC was reported *via* reconstructing standard deviation (±SD) with PC scores and coefficients and plotting against the mean (Warmenhoven et al., 2021). This technique was also used in several other biomechanical studies (O’connor and Bottum, 2009; Almosnino et al., 2013).

In terms of the time-varying biomechanical statistics, the approach of PCA modeling could summarize the variance explanation across time and separate the whole variances into random and deterministic components (O’connor and Bottum, 2009). This was utilized to classify the kinematic and kinetic parameters of knee osteoarthritis (Deluzio et al., 1997) and knee arthroplasty patients (Deluzio et al., 1999) from normal populations. This technique was taken to identify the kinematic response from loaded walking (Lee et al., 2009), knee moments during the squat (Almosnino et al., 2013), and biomechanical performance of jumping (Cushion et al., 2019). Specifically for the GRF, the PCA modeling was taken to discriminate the abnormal walking gait (Muniz and Nadal, 2009) and knee OA (osteoarthritis) gait (Federolf et al., 2013) for clinical assessment, thus showing promising feasibility for the analysis running GRF.

The difference of discrete parameters in GRF (such as LR, first and second peaks) between sexes and speeds was reported in previous studies; however, several time-varying features were still unsolved. To reveal the variations of running GRFs over stance, this study was aimed to develop PCA models to discriminate the vertical and ant–post GRFs across different speeds between male and female runners. The second objective was to investigate the PCs with an explanation of more than 95% variations. The variances in PCs (reconstructed from the 5th and 95th percentiles) will be reported *via* plotting against the mean GRF over stance. The hypothesis was that the male and female recreational runners might present different GRF patterns and magnitude while normalizing to BW across the incremental speeds.

## Materials and Methods

### Participants

A total of 30 recreational runners were recruited to join in this running test, including 15 males (age: 30.2 ± 4.7 years; height: 175.8 ± 2.8 cm; mass: 71.4 ± 3.5 kg) and 15 females (age: 29.7 ± 4.2 years; height: 166.7 ± 3.5 cm; mass: 60.6 ± 2.5 kg). All participants are heel-strike runners and right limb dominant, which was determined as per preferred foot kicking footballs. They all had no injuries or disorders to the musculoskeletal system in the past 6 months before the test. The ethical committee from the Research Academy of Grand Health, Ningbo University in Ningbo, China (no. 2019RAGH1112) approved this study, and the written consent form was obtained with informed objectives, requirements, and procedures.

### Test Protocol

All the running tests were performed on a motorized split belt treadmill with instrumented Bertec force plate (Bertec Corporation, Columbus, OH, United States). Participants were first instructed to run on the treadmill for warm-up and familiarization at a self-selected speed. All participants ran with their own standard running shoes. The GRFs were recorded from the Bertec force plate at a frequency of 1,000 Hz. Running tests under the conditions of four speeds, specifically at 2.7, 3.0, 3.3, and 3.7 m/s, were conducted with an interval of 5-min between each speed to minimize fatigue (Mei et al., 2019; Gao et al., 2020). During the data collection, the treadmill was firstly initiated slowly to runners’ comfortable speed, then adjusted to the test speeds. Once a stable running pattern was observed, the collection of GRF was triggered, and five consecutive steps from the right limb were recorded, then terminated the GRF data collection and slowed down the treadmill. Following the same protocol, 20 steps of GRF data were collected with five steps from each speed condition for each participant. A threshold of 20 N in the vertical GRF was defined as foot strike and toe off (Breine et al., 2019; van Drongelen et al., 2020; Mei et al., 2021), which could avoid noise between treadmill and force plate. The duration of foot strike to toe off was used to compute the contact time of each step.

### Data Process and Statistical Analysis

**FIGURE 1:**
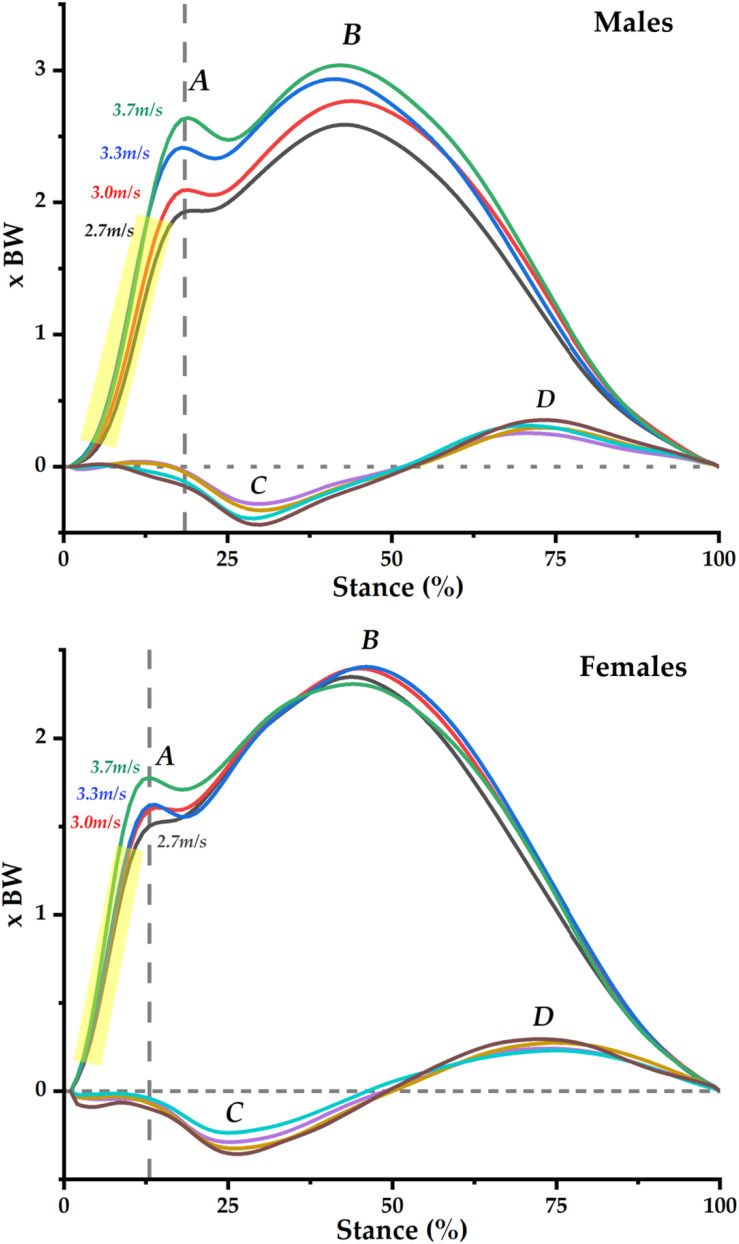
Ground reaction forces in the vertical and anterior–posterior directions across speeds of 2.7, 3.0, 3.3, and 3.7 m/s in male and female runners. First peak **(A)** and second peak **(B)** in the vertical GRF and posterior peak **(C)**, and anterior peak **(D)** in the ant–post GRF. Yellow highlighted is the 20–80% of the first peak for the calculation of vertical average loading rate.

The GRFs were firstly filtered using the Vicon Nexus (Vicon Metrics Ltd., Oxford, United Kingdom) and extracted as CSV files for further data processing and analysis. A pipeline with zero-lag fourth-order low-pass Butterworth filtering was taken to filter the raw GRF data. The GRFs during stance were then interpolated (normalized) into 100 data points to represent the stance percentage (100%). An average of five steps was computed from each runner, and each average was then normalized to BW (body mass × 9.8) for further PCA. As illustrated patterns of GRF from 2.7, 3.0, 3.3, and 3.7 m/s (Figure 1), the vertical GRF includes a first peak (***A***), a second peak (***B***), and impact transient between the first and second peak. The GRF in the ant–post direction includes a posterior (braking) peak (***C***) and an anterior (propulsion) peak (***D***). The med–lat GRF was not the main objective from the current study, which was discussed in the section “Introduction.” Furthermore, the med–lat GRF was reported in Supplementary Material for reference of potential interest, and the raw data were available from our online repository.^1^

The discrete values of vertical average loading rate (VALR) from each runner were calculated following an established protocol (Milner et al., 2006; Ueda et al., 2016), from 20 to 80% of the time to the first peak (***A*** as highlighted in Figure 1). Specifically, VALR = [*F*_80%_ - *F*_20%_] / *[t*_80%_ - *t*_20%_]. The *F*_80%_ and *t*_80%_ represent the force magnitude and time, respectively, till the 80% of the first peak, and the *F*_20%_ and *t*_20%_ represent the force magnitude and time, respectively, till the 20% of the first peak.

In terms of the statistical analysis of the discrete variables (VALR), the independent sample *t*-test was conducted to check the difference between male and female runners at the same speed condition. The one-way repeated measures analysis of variance (one-tail) with Tukey *post hoc* test was taken to analyze the difference (higher/lower or larger/smaller) across four speeds in male and female participants. All the statistical analyses were performed using the SPSS v 21 (IBM Corp., Armonk, NY, United States). The significance level was set at *a* < 0.05.

An advanced statistical analysis using PCA was conducted to reduce the high dimensionality in the data matrices and project onto PCs (Lever et al., 2017), thus extracting the key features of variation in the GRFs.

(1) $$\left[\begin{array}{ccc} {\text{x}}_{1}^{1} & {\text{x}}_{1}^{2}\,\cdots \,{\text{x}}_{1}^{99} & {\text{x}}_{1}^{100}\\ \vdots & \ddots \,\ddots \,\ddots & \vdots \\ {\text{x}}_{m}^{1} & {\text{x}}_{m}^{2}\,\cdots \,{\text{x}}_{m}^{99} & {\text{x}}_{m}^{100} \end{array}\right]=\left[\begin{array}{ccc} {\text{z}}_{1}^{1} & {\text{z}}_{1}^{2} & {\text{z}}_{1}^{3}\,{\text{z}}_{1}^{4}\\ \vdots & \ddots & \ddots \,\vdots \\ {\text{z}}_{m}^{1} & {\text{z}}_{m}^{2} & {\text{z}}_{m}^{3}\,{\text{z}}_{m}^{4} \end{array}\right]\,\begin{array}{c} {\text{T}}_{1}^{2}\\ \vdots \\ {\text{T}}_{m}^{2} \end{array}\,\begin{array}{c} {\text{Q}}_{1}\\ \vdots \\ {\text{Q}}_{m} \end{array}$$

As presented in Equation (1), the original matrices (***X** = **x**^1^, **x**^2^, **x**^3^, … **x**^99^, **x**^100^*) × *m* were orthogonally transformed into uncorrelated PCs (***Z** = **z**^1^, **z**^2^, **z**^3^*, …, ***z**^*p*^*) (***p*** < 100), corresponding loading vectors (***T**^2^ = **T**_1_, **T**_2_, **T**_3_, …**T**_*m*_*) and residuals (*Q*), which was defined as ***Z** = **X*** ^∗^ ***T**^2^* (Deluzio et al., 1997). Specifically, the *m* equals 60 (4 × 15 × 100 matrices) for PCA modeling of the four speeds in male and female runners.

This study mainly accounted for the main variations in the first *k* (*k* = 4 in this study) PCs (*z^1^, z^2^, z^3^, & z^4^*), which explained more than 95% of variances. The first four PCs in the vertical and ant–post GRFs were reconstructed with the scores and coefficients of each PC by calculating the plus/minus SD to represent the Upper (plus SD) and Lower (minus SD) from the PCA modeling (Lee et al., 2009; O’connor and Bottum, 2009; Brandon et al., 2013; Warmenhoven et al., 2021). The reconstructed GRF from the Upper and Lower limits of each PC were then plotted against the mean for visualizing the key features. Specifically, the line with symbols “+” and “▼” represents upper (plus SD) and lower (minus SD) limits for each PC. All the matrices of GRFs ran the PCA modeling in the MATLAB software (R2019a, The MathWorks Inc., MA, United States).

## Results

### Gender Difference

**FIGURE 2:**
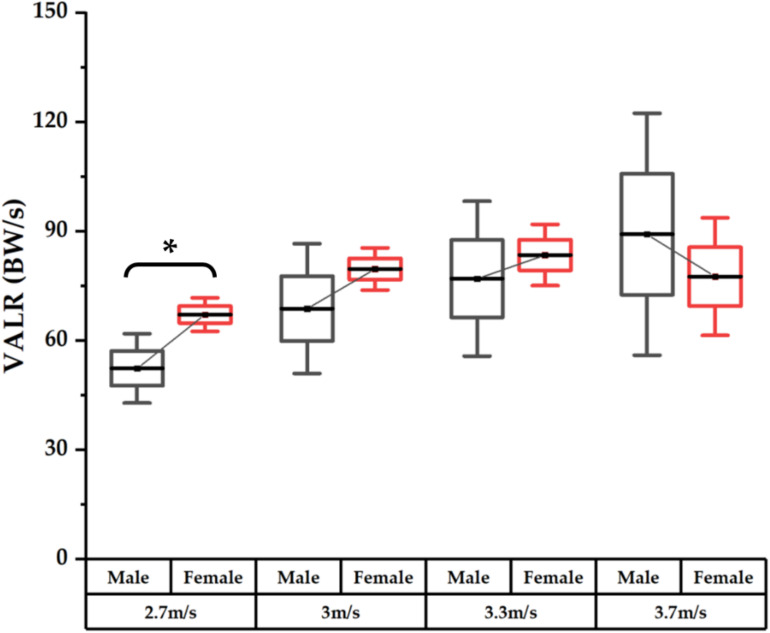
Comparison of vertical average loading rates (VALRs) between male and female runners across different speeds with mean, 25–75th percentile, and SD values and highlighted significance with an asterisk (*).

As presented in Figure 2, male runners exhibited significantly (*p* < 0.001) smaller VALR loadings than female runners during running at 2.7 m/s, whereas no significance was observed across the running speeds of 3.0, 3.3, and 3.7 m/s.

### Speeds

**FIGURE 3:**
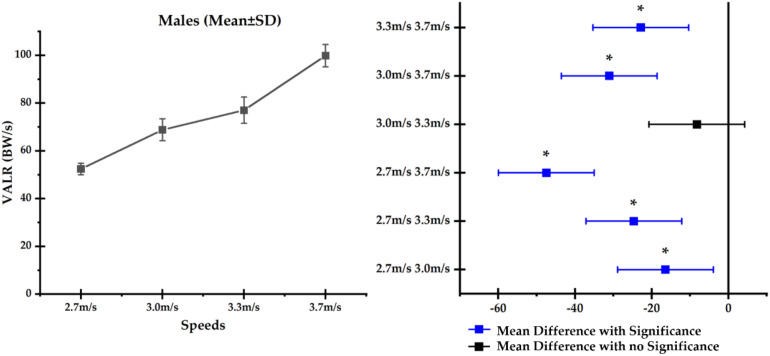
Comparison of vertical average loading rates (VALRs) across different running speeds (mean ± SD), **(left)** and mean difference **(right)** in male runners with significance highlighted in blue and asterisk (*).

Among male runners, a significant difference was observed between 2.7 and 3.0 m/s (*p* = 0.005), 2.7 and 3.3 m/s (*p* < 0.001), 2.7 and 3.7 m/s (*p* < 0.001), 3.0 and 3.7 m/s (*p* < 0.001), and 3.3 and 3.7 m/s (*p* < 0.001), apart from 3.0 and 3.3 m/s (*p* = 0.306). The mean differences between each speed condition and highlighted significance (asterisk, ^∗^) are reported in Figure 3.

**FIGURE 4:**
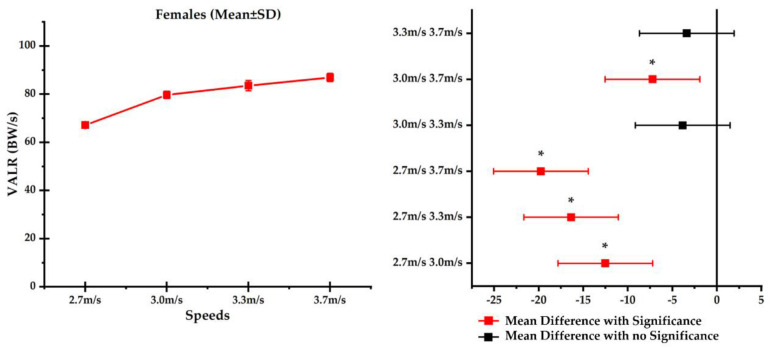
Comparison of vertical average loading rates (VALRs) across different running speeds (mean ± SD), **(left)** and mean difference **(right)** in female runners with significance highlighted in red and asterisk (*).

In terms of the difference while running with different speeds in the female runners, a significant difference was observed between 2.7 and 3.0 m/s (*p* < 0.001), 2.7 and 3.3 m/s (*p* < 0.001), 2.7 and 3.7 m/s (*p* < 0.001), and 3.0 and 3.7 m/s (*p* < 0.003), apart from 3.0 and 3.3 m/s (*p* = 0.229) and 3.3 and 3.7 m/s (*p* = 0.334). The mean differences between each speed condition and highlighted significance (asterisk, ^∗^) are reported in Figure 4.

### Principal Component Analysis of Ground Reaction Force Across Speeds

**FIGURE 5:**
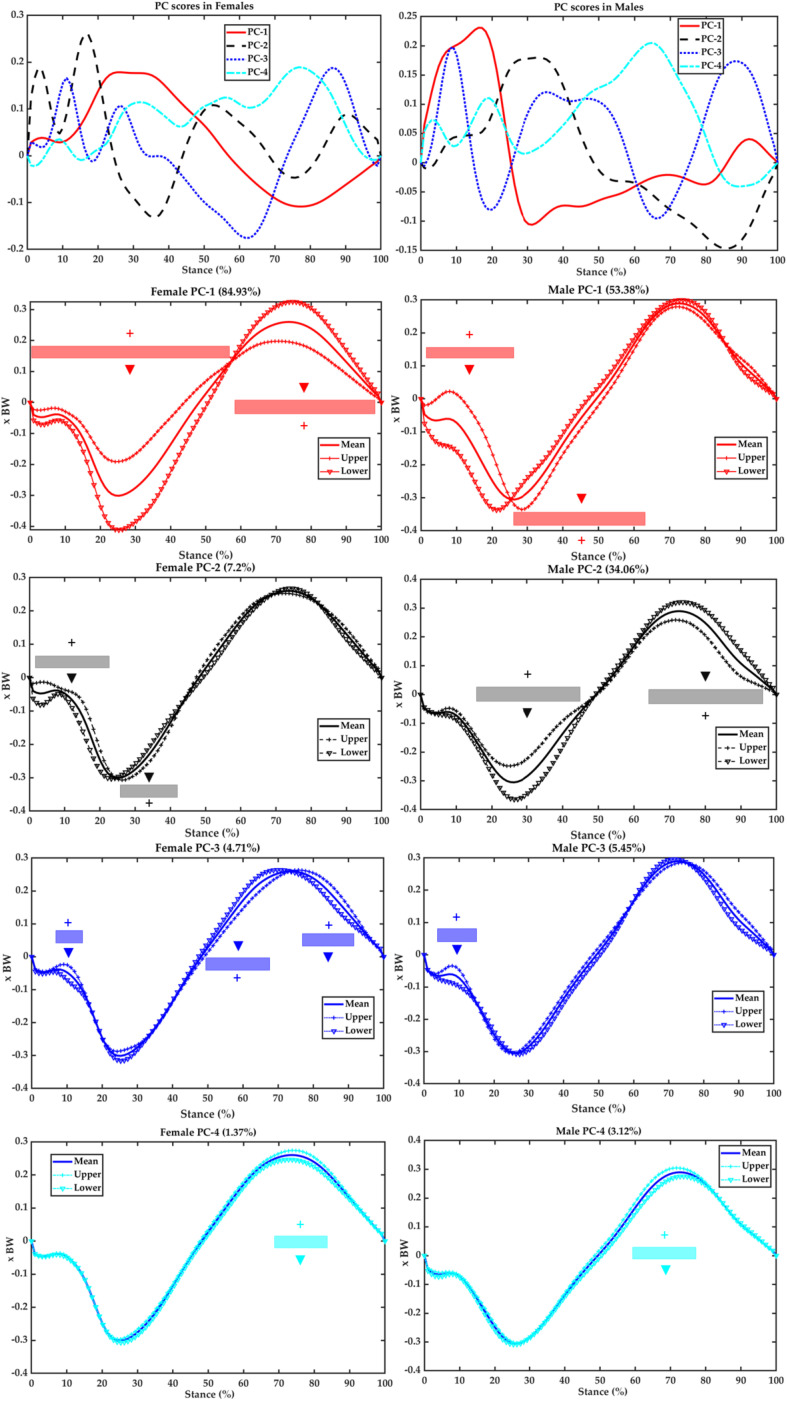
Scores of the first four PCs (first row) and variances of PC1 (second row in red), PC2 (third row in black), PC3 (fourth row in blue), and PC4 (fifth row in cyan) against the mean ant–post GRF of the female **(left)** and male **(right)** runners. Upper (plus SD) limit (with symbol “+”) and lower (minus SD) limit (with symbol “▼”) highlight the contribution of plus/minus of the scores and coefficients for this PC.

After the PCA modeling of the ant–post GRF (Figure 5), the PC1 in females explains 84.93% of variations, specifically during 2–57% of the upper limit over the lower limit and 58–99% of the lower limit over the upper limit. The PC1 in males explains 53.38% of variations during 2–25% of the upper limit over the lower limit and during 26–63% of the lower limit over the upper limit. The PC2 accounts for 7.2% in females during 2–22% of the upper over the lower limit and 26–43% of the lower over the upper limit, and the PC2 explains 34.06% in males during 16–46% of the upper over the lower limit and 62–96% of the lower over the upper limit. The PC3 explains 4.71% in females during 8–14 and 77–93% of the upper over the lower limit and 49–69% of the lower over the upper limit and accounts for 5.45% in males during 5–14 and 81–98% of the upper over the lower limit. The PC4 explains variances of 1.37% (females) during 67–83% and 3.12% (males) during 60–78% with the upper limit over the lower limit.

As included in the Supplementary Document, the mean GRF in the ant–post, med–lat, and vertical directions are illustrated in Supplementary Figure 1. Extra information of the percentage of variances explained and PC1 against PC2 are included in Supplementary Figures 2, 3.

**FIGURE 6:**
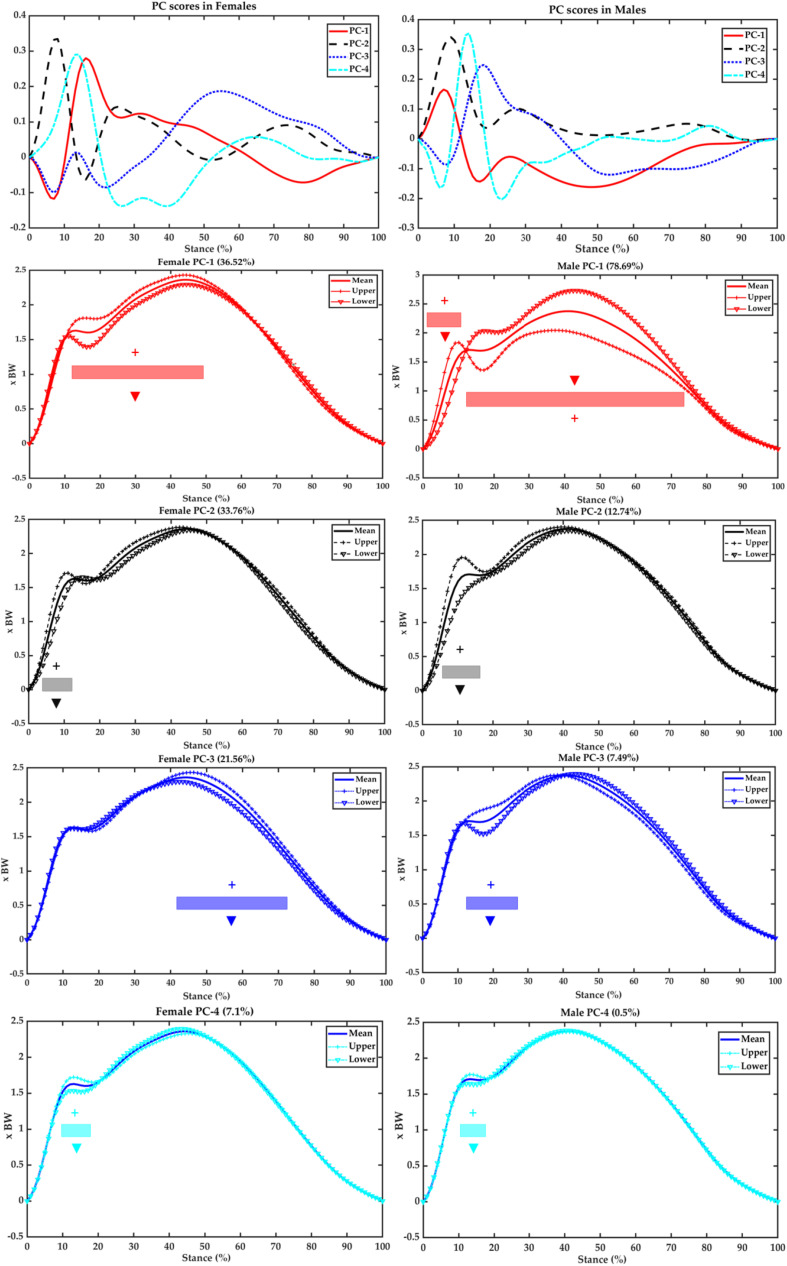
Scores of the first four PCs (first row) and variances of PC1 (second row in red), PC2 (third row in black), PC3 (fourth row in blue), and PC4 (fifth row in cyan) against the mean vertical GRF of the female **(left)** and male **(right)** runners. Upper (plus SD) limit (with symbol “+”) and lower (minus SD) limit (with symbol “▼”) highlight the contribution of plus/minus of the scores and coefficients for this PC.

As presented in Figure 6 of PCA modeling the vertical GRF, the PC1 in females accounts for 36.52% and explains variations of 78.69% in males. Specifically, the females vary during 12–50% in the upper over the lower limit compared with the mean vertical GRF, whereas the upper over the lower limits vary during 0–12%, which are opposite with the lower over the upper limit from 13 to 76% in males. The PC2 in females and males explains variations of 33.76 and 12.74%, which are mainly located during the first peak in both sexes, specifically during 4–12% in females and 5–16% in males. The PC3 accounts for 21.56% (females) and 7.49% (males) variations, and females showed variations in the second peak during push-off from 42 to 74%, whereas males presented variations during transient from 13 to 28%. The PC4 explains variances of 7.1% (in females) during 9–18% and 0.5% (in males) during 11–17% in the first peak.

## Discussion

The primary objective of the current study was to develop PC modeling of GRFs during running with incremental speeds between female and male runners and further reveal the main modes (regions) of variations in the ant–post and vertical GRFs. Typically for the discrete variables, the VALR increased as speeding up (2.7, 3.0, and 3.3 m/s) in both sexes, and females had higher VALR than males apart from the speed of 3.7 m/s. These are similar to literature works that variables in the GRF increased as running velocity increased (Van den Berghe et al., 2019; Williams et al., 2020). Furthermore, it was observed that the ant–post GRF varies in the PC1 of magnitude variances in female runners, whereas males vary in both PC1 (timing of braking peak) and PC2 (magnitude). The PC1 (transient and first peak) and PC2 (LR) of vertical GRF explained a similar percentage of variations in females, whereas males had PC1 (LR and second peak combined) accounted for the most variations.

Running GRF is related to impact shock, loading accumulation, and even stress syndrome in the lower extremity (Lieberman et al., 2010; Breine et al., 2019). While comparing the VALRs in the males and females across the four speeds, significance was observed only during 2.7 m/s without significance in other speeds. This might be explained that female and male runners showed no consistent difference during running-related agility tasks in a sex-based comparative study (Nakamura et al., 2012). The VALRs increase consecutively as incrementing running speeds, apart from a reduction of VALR in females during 3.7 m/s. This difference suggests that females and males may present an altered running strategy or pattern (Clermont et al., 2019) and neuromuscular performance (Huston and Wojtys, 1996). This should be noted on the fact that both female and male runners in this study are recreational runners, presenting higher variability compared with competitive and elite runners with consistent running gait patterns (Cavanagh et al., 1977; Clermont et al., 2019; Preece et al., 2019).

While running under incremental speed conditions, the values of the mean difference increased greatly. Apart from that, the 3.0–3.3 m/s running showed a subtle difference; it may be due to that this speed range (3.0–3.3 m/s, around 11–12 km/h) is commonly adopted self-selected running speeds in the cohort of recreational runners (Hoenig et al., 2019; Mei et al., 2019); thus, the comparison of VALR shows no significant difference. The LR is discretely viable, which was calculated from 20 to 80% of the first peak in the heel striking running GRF (Milner et al., 2006; Ueda et al., 2016). Recent studies on running-related injuries reported that the accumulation of impact loads might lead to increased injury risks (Bertelsen et al., 2017; Mei et al., 2019; Backes et al., 2020). Furthermore, the increases of GRF parameters may not be a direct indicator of tibia bone loads or overuse injury risks (Matijevich et al., 2019), as this may link to other intrinsic muscular contributions and mechanical alignment (Mei et al., 2019). Thus, the time-varying GRFs over stance are utilized to estimate the load accumulation. In addition, the collection of GRF requires force plates either during overground or instrumented treadmill running. Both test conditions show consistency in measuring the vertical GRF with stable landing patterns across different running speeds (Kluitenberg et al., 2012; Van Hooren et al., 2020).

In terms of the PCA modeling of ant–post GRF, females presented variation in the magnitudes of braking and propulsive force (PC1) over the timing of braking propulsion (PC2, PC3, and PC4). In contrast, males had higher variation in the timing of braking-propulsion transition (PC1) over magnitudes of braking and propulsive forces (PC2, PC3, and PC4). This may be explained that female and male runners used different brake-propulsion strategies as incrementing speeds (Clermont et al., 2019). Specifically, female runners may have a higher variation or less stable running patterns (Clermont et al., 2019), whereas male runners may vary in the time of propulsion but still higher propulsive forces (Bazuelo-Ruiz et al., 2018). This was reported to link with running performance *via* comparative analysis of level-based runners (Preece et al., 2019).

The difference of variations in the vertical GRF between female and male runners was similar with ant–post GRF. Typically, in the females, the transient between first and second peaks (PC1) explained the principal variation over LR region (PC2), second peak (push-off) region (PC3), and first peak region (PC4). On the contrary, the second peak (push-off) force (PC1) and LR (PC2) took over the transient (PC3) and first peak (PC4) in male runners. Evidence was that females and males might respond differently as running speed incrementing (Breine et al., 2019; Clermont et al., 2019). In specifics, the females showed variations in the impact absorption and force output, whereas males changed the timing of force output. This was similar to the discrepancy discussed earlier in the ant–post GRF, which is related to running performance (Preece et al., 2019). Further explanation was that gait patterns adjusted and adapted as speeds increasing in both sexes, particularly the vertical GRF as key biomechanical parameters (Pavei et al., 2019). Another key message that should be noted was the variances explained in the PC2 in females (33.76%) and males (12.74%) in the LR region, which suggested that female runners had higher variations of LR as speeding up. This was consistent with findings reported from a recent review that female runners had a higher risk of bone stress injuries than male runners, which related to LRs from landing impact (Hollander et al., 2021).

Several recent studies were performed to estimate the GRF from wearables fused with machine learning or artificial intelligence techniques (Shahabpoor and Pavic, 2017; Hendry et al., 2020; Jiang et al., 2020). This was aimed to address the challenge of high dynamics or environmental variations from the lab to in-field measurement. An acceptable agreement of the GRF measured from the instrumented treadmill and overground running was found in both female and male runners, especially the vertical GRF (Kluitenberg et al., 2012). However, the inconsistency was also reported due to the factors of friction between the belt and surface of force plate and motor pulling (Willems and Gosseye, 2013). Specifically, the split dual-belt motorized treadmill in this study might affect the step width, which was sensitive to the med–lat GRF (McClay and Cavanagh, 1994). In this study, the med–lat GRF was not a primary objective and reported in Supplementary Material. In terms of the vertical GRF and ant–post GRF, the difference of running on a treadmill *versus* overground was reported with reduced peak propulsive force and impact loadings (Willems and Gosseye, 2013; Van Hooren et al., 2020). In contrast, multiple steps of measurement on the treadmill were suggested to represent the GRF magnitude and pattern (Kluitenberg et al., 2012; Willems and Gosseye, 2013), and in the current study, five consecutive steps were measured to address this concern. Furthermore, a PCA modeling was used in the current study to extract the key variances of GRFs. The findings revealed the main features (eigenvectors and eigenvalues) of variations between sexes, particularly in the ant–post and vertical directions. These may have practical implications for the prevention of sex-specific running-related injuries. The information would be integrated with GRFs estimated from wearables for the calibration of accelerometer signals. It could be further implemented as a training dataset in the machine learning models for the rapid and accurate prediction of running GRFs across different speeds.

Several limitations should be considered while acknowledging the findings from the current study. Firstly, the running footwear was not controlled in this study, as we were aiming to collect the running GRF response from variate footwear conditions. This is to follow the “real-scenario” in field context while using machine learning models, whereas it should be noted that the footwear condition was an issue that may affect the GRF (Logan et al., 2010). Secondly, the motion capture data were not collected, as the female and male runners may have different running patterns, although all heel strikes were observed from the vertical GRF with first peak and second peak. A subtle different foot strike angles may affect the ant–post and vertical GRFs. Lastly, we used the same incremental speed for both female and male runners without considering the potential sex preference of absolute and relative optimal running speeds, which shall be a topic for future studies. To sum up all limitations concerning the current study, future research shall consider synchronously collecting the kinematics, GRFs, and wearable signals to develop machine learning models based on “ground-truth” dataset.

## Conclusion

This study developed PCA models to extract the main features of variances in the GRFs of female and male runners across different speeds. Female and male runners behaved differently in the ant–post and vertical GRFs. In specifics, the females mainly varied in the magnitude of braking and propulsive forces, whereas the male runners varied in the timing of propulsion in the ant–post GRF. In the vertical GRF, the female runners varied in the transient between first and second peaks and LR, whereas the males varied in the LR and active push-off forces (second peak). Knowledge reported in the current study may have implications for the prevention of sex-specific running-related injuries. It could be further integrated with wearables signals for the in-field prediction and estimation of impact loadings and GRFs.

## Data Availability Statement

The original contributions presented in the study are included in the article/Supplementary Material, further inquiries can be directed to the corresponding authors.

## Ethics Statement

The studies involving human participants were reviewed and approved by Research Academy of Grand Health, Ningbo University in Ningbo, China (No. 2019RAGH1112). The patients/participants provided their written informed consent to participate in this study.

## Author Contributions

LY, QM, JF, and YG: conceptualization. LY, QM, and LX: methodology. QM and JF: software. NIM and YG: validation. LY, QM, LX, WL, and NIM: formal analysis. LY, QM, and NIM: investigation. YG: resources. QM, LX, and WL: data curation. LY, QM, and LX: writing—original draft preparation. JF and YG: writing—review and editing. LY and QM: visualization. JF and YG: supervision. YG: project administration. QM and YG: funding acquisition. All authors have read and agreed to the published version of the manuscript.

## Conflict of Interest

The authors declare that the research was conducted in the absence of any commercial or financial relationships that could be construed as a potential conflict of interest.

## Footnotes

1 https://simtk.org/projects/longdistrun
