# Supplementary material for: Principal Component Analysis of the Running Ground Reaction Forces With Different Speeds
Source: Front Bioeng Biotechnol. 2021 Mar 25;9:629809. doi: 10.3389/fbioe.2021.629809 (PMC8026898; doi:10.3389/fbioe.2021.629809)
Supplement: Supplementary file 1 [file Data_Sheet_1.docx]

These supplementary documents include the mean ground reaction forces in the ant-post, vertical, and med-lat directions (**Figure A1**), the percentage of variations and accumulative explanation (**Figure A2**), and the PC1 against PC2 of the components (**Figure A3**).


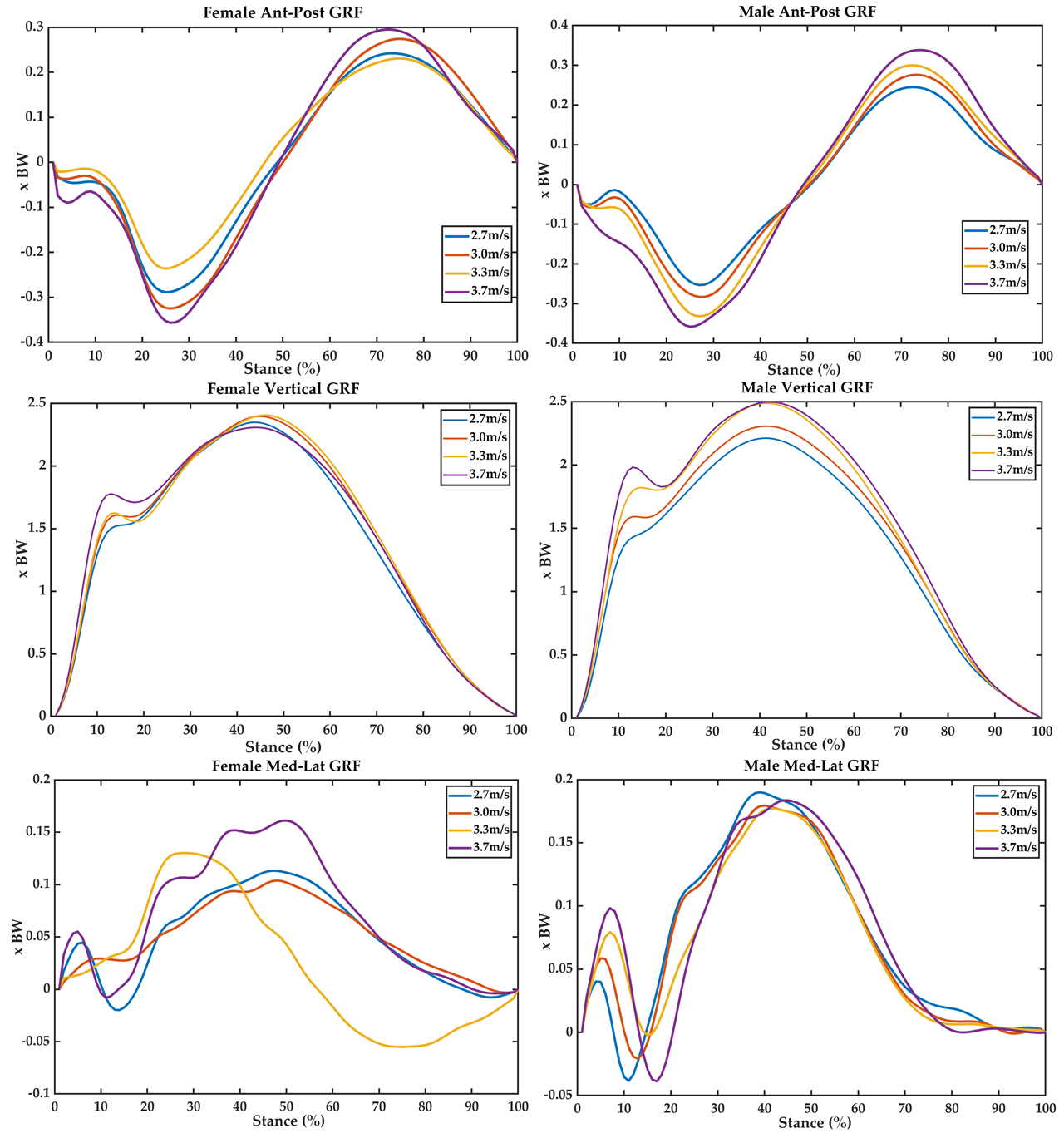


**Figure A1.** Mean GRFs in the female and male runners across the incremental speeds
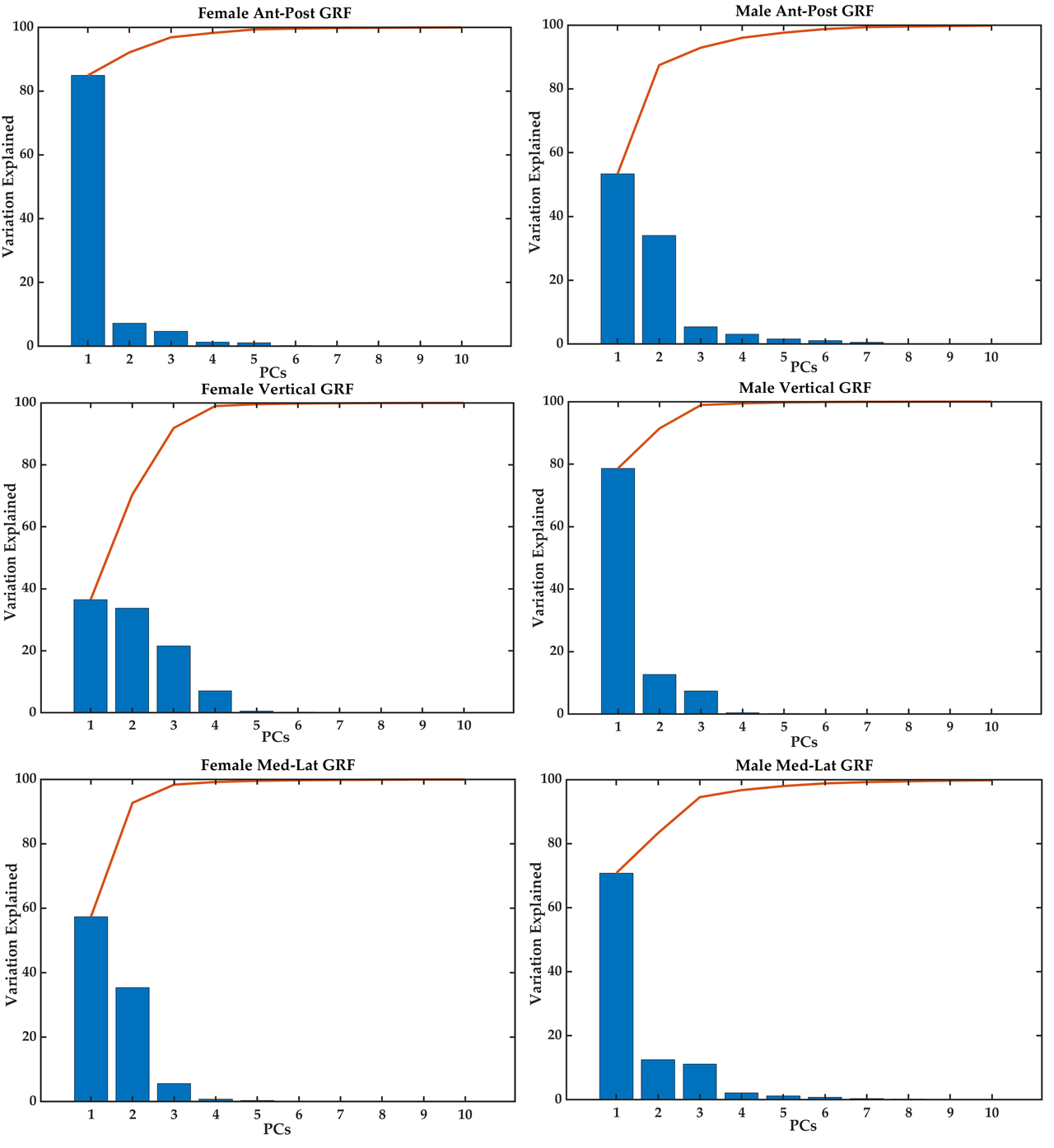


**Figure A2.** Percentage of variances explained in the first 10PCs and accumulative variations


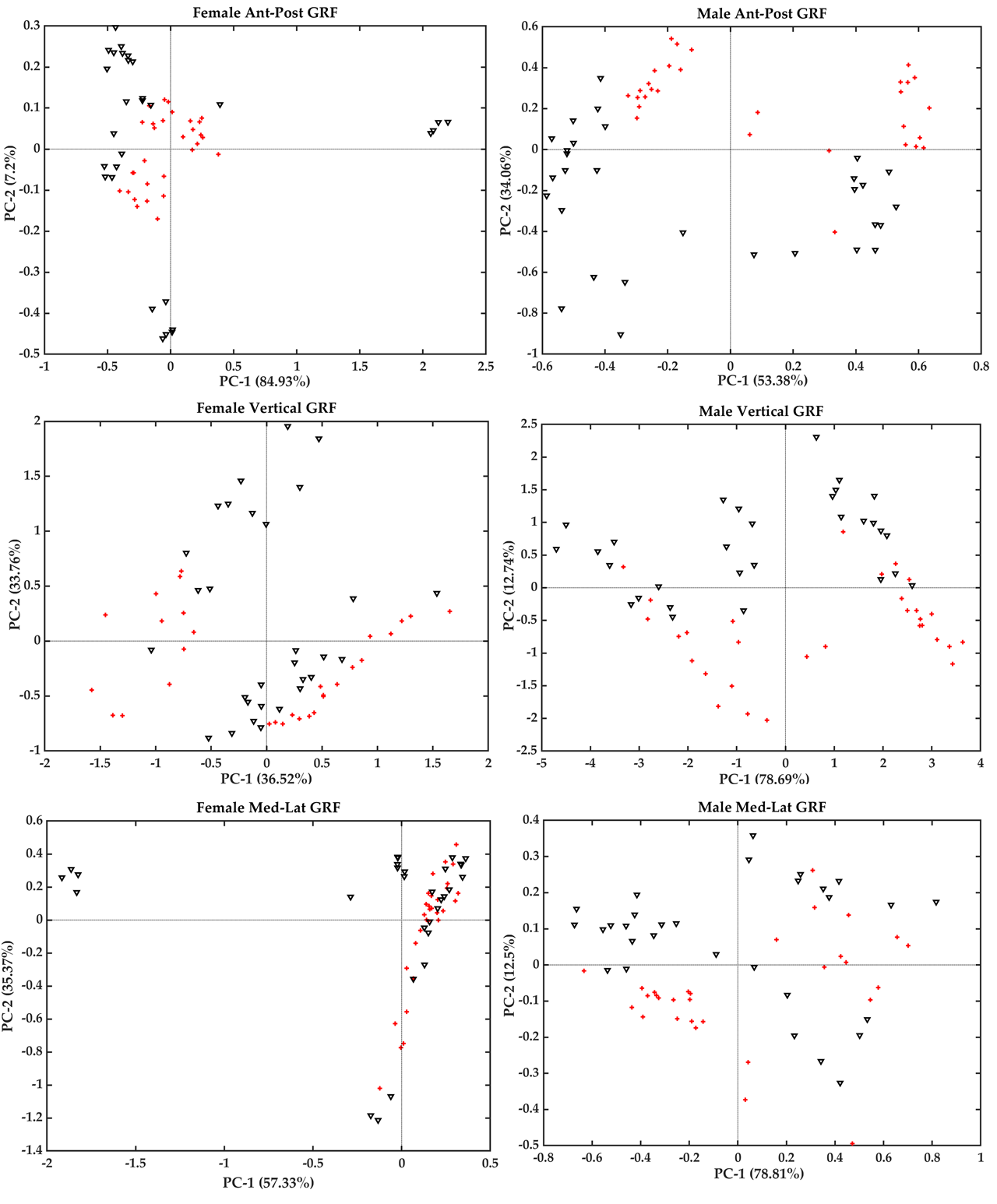


**Figure A3.** Illustration of PC1 against PC2 in the PCA modelling of GRFs
